# Epigallocatechin Gallate Attenuates Proliferation and Oxidative Stress in Human Vascular Smooth Muscle Cells Induced by Interleukin-1**β** via Heme Oxygenase-1

**DOI:** 10.1155/2014/523684

**Published:** 2014-09-07

**Authors:** Po-Len Liu, Jung-Tung Liu, Hsuan-Fu Kuo, Inn-Wen Chong, Chong-Chao Hsieh

**Affiliations:** ^1^Department of Respiratory Therapy, College of Medicine, and Department of Medical Research, Translational Research Center, Kaohsiung Medical University, Kaohsiung 807, Taiwan; ^2^School of Medicine, College of Medicine, Chung Shan Medical University, Taichung 402, Taiwan; ^3^Department of Neurosurgery, Chung Shan Medical University Hospital, Taichung 402, Taiwan; ^4^Department of Internal Medicine, Kaohsiung Municipal Ta-Tung Hospital, Kaohsiung Medical University Hospital, Kaohsiung Medical University, Kaohsiung 801, Taiwan; ^5^Department of Internal Medicine, Department of Chest Surgery, Division of Cardiovascular Surgery and Department of Surgery, Kaohsiung Medical University Hospital, Kaohsiung 807, Taiwan

## Abstract

Proliferation of vascular smooth muscle cells (VSMCs) triggered by inflammatory stimuli and oxidative stress contributes importantly to atherogenesis. The association of green tea consumption with cardiovascular protection has been well documented in epidemiological observations, however, the underlying mechanisms remain unclear. This study aimed to elucidate the effects of the most active green tea catechin derivative, (−)-epigallocatechin-3-gallate (EGCG), in human aortic smooth muscle cells (HASMCs), focusing particularly on the role of a potent anti-inflammatory and antioxidative enzyme heme oxygenase-1 (HO-1). We found that pretreatment of EGCG dose- and time-dependently induced HO-1 protein levels in HASMCs. EGCG inhibited interleukin- (IL-)1*β*-induced HASMC proliferation and oxidative stress in a dose-dependent manner. The HO-1 inducer CoPPIX decreased IL-1*β*-induced cell proliferation, whereas the HO-1 enzyme inhibitor ZnPPIX significantly reversed EGCG-caused growth inhibition in IL-1*β*-treated HASMCs. At the molecular level, EGCG treatment significantly activated nuclear factor erythroid-2-related factor (Nrf2) transcription activities. These results suggest that EGCG might serve as a complementary and alternative medicine in the treatment of these pathologies by inducing HO-1 expression and subsequently decreasing VSMC proliferation.

## 1. Introduction

Atherosclerosis, the major pathological condition for most stroke syndromes, has been the intense focus of basic, clinical, and epidemiological studies. These studies have direct bearing on the prevention of atherothrombotic brain infarction [1–3]. Atherosclerosis is typically multifactorial, most often dependent on oxidative stress-related risk factors such as hypercholesterolemia, diabetes, smoking, hypertension, and obesity. Complications of atherosclerosis remain the leading cause of morbidity and mortality in industrialized countries [4–6].

The migration of smooth muscle cells into the intima, followed by their proliferation, is a central theme of atherosclerosis and restenosis. Various studies suggest that these events are preceded and accompanied by oxidative stress and inflammation [7–9]. Cytokines of the interleukin-1 (IL-1*β*) family play a pivotal role in regulating inflammatory responses, and extensive studies have been performed to determine whether IL-1*β* modifies the inflammatory response [10, 11]. IL-1*β* induces a substantial increase in the expression of inflammatory factors by vascular smooth muscle cells (VSMCs), and these factors promote leukocyte recruitment and infiltration into the arterial wall and stimulate VSMC proliferation [12, 13]. Recent studies have shown increased levels of IL-1*β* in human atherosclerotic lesions [14].

**Figure 1:**
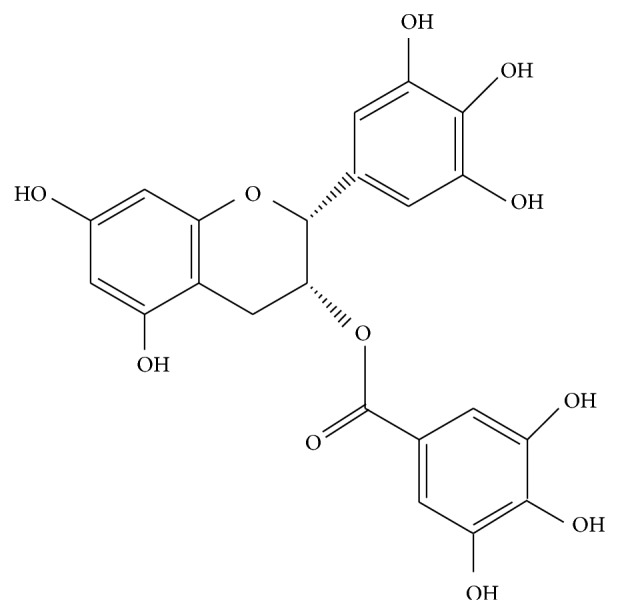
Chemical structure of (−)-epigallocatechin-3-gallate (EGCG), the most abundant and most active catechin derivative from green tea.

The consumption of green tea has attracted attention because of its beneficial effects on health. The association of green tea consumption with cardiovascular protection has been well documented in epidemiological observations [15]. The polyphenolic catechins found in green tea include (−)-epigallocatechin-3-gallate (EGCG) (Figure 1), (−)-epicatechin-3-gallate (ECG), (−)-epigallocatechin (EGC), and epicatechin (EC). These phenolic compounds appear to interfere with the molecular processes underlying the initiation, progression, and rupture of atherosclerotic plaques [16]. EGCG is the most abundant and most active catechin derivative and has been reported to exhibit both anti-inflammatory and antiatherogenic properties in experimental studies associated with its antioxidative activity [17].

Heme oxygenase-1 (HO-1) is a member of the heat shock protein family. Its expression is triggered by diverse stress inducing stimuli including hypoxia, heavy metals, UV radiation, and reactive oxygen species (ROS) [18–20]. HO-1 can catalyze heme to carbon monoxide and bilirubin with a concurrent release of iron. The physiologic functions of HO-1 entail antioxidation, anti-inflammation, antiproliferation, and antiapoptosis effects [21]. Recent studies suggest that plant-derived chemical substances may act as inducers of the response protein HO-1 and can maximize the intrinsic antioxidant potential of compounds [22].

In this study, we analyzed the potency of EGCG as an inducer of HO-1 expression in human aortic smooth muscle cells (HASMCs) and explored whether this contributed to the protective effects of this polyphenolic compound against smooth muscle cell proliferation and oxidative stress.

## 2. Materials and Methods

### 2.1. Cell Culture

HASMCs (Cascade Biologics, OR, USA) were grown and passaged as described previously [23, 24]. Cells were used at passages 3–8. The purity of HASMC cultures was verified by immunostaining with a monoclonal antibody directed against smooth-muscle-specific *α*-actin (R&D Systems, MN, USA). Before treatment or stimulation with reagents, the cells were serum starved for 24 h.

### 2.2. Western Blot Analysis

Total cell lysates were prepared in lysis buffer (20 mM Tris-HCl, 150 mM NaCl, 1 mM EDTA, 1 mM EGTA, 1% Triton, 2.5 mM sodium pyrophosphate, 1 mM glycerophosphate, 1 mM Na_3_VO_4_, 1 *μ*g/mL leupeptin, and 1 mM PMSF, pH 7.5). The protein concentration was determined with a Bio-Rad protein assay reagent (BIO-RAD, CA, USA) and the samples were stored at −70°C [25].

Western blot analysis was used to determine the changes in cell levels of HO-1 in HASMCs. Proteins were separated by sodium dodecyl sulfate-polyacrylamide gel electrophoresis (SDS-PAGE) and transferred to polyvinylidene fluoride (PVDF) membrane, and then the membranes were blocked with 5% milk. The PVDF membranes were probed with goat anti-HO-1 antibody (R&D Systems, MN, USA) at 1 : 1000 and then incubated with horseradish peroxidase- (HRP-) conjugated secondary antibodies and the proteins were visualized with an enhanced chemiluminescence detection kit (Amersham Biosciences, NJ, USA). Mouse anti-*β*-actin (Labvision/NeoMarkers, CA, USA) antibodies were used as loading controls. Protein expression levels will be quantified as optical densities, using ImageQuant software [26].

### 2.3. BrdU Incorporation Assay for Cell Proliferation

HASMCs were seeded on 96-well plates at a density of 1 × 10^4^ cells/well. Cells were incubated with various concentrations of EGCG for 24 h and then stimulated with 50 ng/mL of IL-1*β* for 8 h. DNA synthesis was measured using a 5-bromo-20-deoxyuridine (BrdU) cell proliferation kit (Calbiochem, Darmstadt, Germany). Briefly, after 4 h of incubation with IL-1*β*, BrdU labeling solution (BrdU concentration: 10 *μ*M) was added to the cells and incubated for another 4 h. After removing the culture medium, the cells were fixed and nucleases were added to partially digest cellular DNA. Anti-BrdU antibody was then added before adding the mouse IgG-peroxidase conjugate. The signal was developed with tetramethylbenzidine solution in the dark. A spectrophotometric plate reader to measure absorbance was used at dual wavelengths of 450 nm/595 nm [27].

### 2.4. Cell Cycle Analyses with Flow Cytometry

Analysis of the DNA content and the movement of the cells through the mitotic cycle were performed by flow cytometry 24 h after cell stimulation. HASMCs were harvested and fixed in 70% ethanol; then cells were washed with ice-cold PBS and incubated with RNAase (Sigma, MO, USA) and propidium iodide (Sigma, MO, USA). Cell cycle phase analysis was performed by flow cytometry using a FACScan (Becton Dickinson, NJ, USA), and the percentage of cells in different phases of the cell cycle was analyzed using ModFitLT software (BD, NJ, USA) [28].

### 2.5. Detection of ROS Production

The effect of EGCG on ROS production in HASMCs was determined by a fluorometric assay using DCFH-DA as the probe. This method is based on the oxidation by H_2_O_2_ of nonfluorescent DCFH-DA to fluorescent 2′,7′-dichlorofluorescin (DCF). Confluent HASMCs (10^4^ cells/well) in 48-well plates will be pretreated with various concentrations of EGCG. The cells were washed with HBSS, then HBSS containing 10 *μ*M DCFH-DA was added, and incubation continued for 45 min at 37°C. The fluorescence intensity (relative fluorescence units) was measured at 485 nm excitation and 530 nm emission using a Fluorescence Microplate Reader [29].

### 2.6. Measurement of Glutathione (GSH) and Thiobarbituric Acid Reactive Substances (TBARS) Levels

Intracellular GSH levels were measured using a colorimetric assay (Bioxytech GSH-400; OxisResearch, Portland, OR). Briefly, cells were washed with PBS, and metaphosphoric acid (5%) was added to the cells, which were then scraped off. The mixture was centrifuged at 3,000 g for 5 min at 4°C, and the supernatant was measured at 400 nm after a chemical reaction with reagent R1 (4-chloro-1-methyl-7-trifluromethyl-quinolinium methylsulfate) and reagent R2 (30% NaOH). A known concentration of GSH was used to generate a standard curve [30].

Lipid peroxidation was quantified by measuring TBARS by spectrophotometric assay (Beckman Coulter, DU 640 spectrophotometer, Germany) according to a previous study. The level of lipid peroxides, expressed as nanomoles of malondialdehyde (MDA) per milligram of protein, was calculated from the absorbance at 532 nm using tetraethoxypropane (TEP) as an external standard [30].

### 2.7. Nuclear Extract Preparation and Electrophoretic Mobility Shift Assay (EMSA)

Nuclear protein extracts were prepared as previously described [19]. In brief, cells were scraped off the plates in an ice-cold HEPES buffer. After centrifugation at 300 g for 10 minutes at 4°C, the cells were solubilized with the above buffer with 0.1% Triton X-100 and centrifuged at 12,000 g for 10 minutes at 4°C. The nuclear pellets were resuspended in a buffer (10 mM HEPES, pH 7.9, 1.5 mM MgCl_2_, 0.42 M NaCl, 1 mM DTT, 0.2 mM EDTA, 1.0 mM PMSF, 25% glycerol, 0.5 mM PMSF, 2 *μ*g/mL aprotinin, 2 *μ*g/mL pepstatin, and 2 *μ*g/mL leupeptin), incubated for 30 minutes at 4°C, and centrifuged at 15,000 g for 30 minutes at 4°C.

The probe for nuclear factor E2-related factor-2 (Nrf2) gel shift assays was a synthetic double-stranded oligonucleotide (5′-AGA TTT TGC TGA GTC ACC AGT CCC-3′) containing the consensus Nrf2 binding site with antioxidant response element (ARE) [31]. For EMSA, doubled-stranded DNA was end-labeled with dig-dUTP using terminal transferase (Roche, Germany). The DNA-binding reaction was performed with 5 *μ*g nuclear proteins and 0.8 ng digoxin-labeled oligonucleotide at room temperature for 15 minutes. Nuclear extract-oligonucleotide mixtures were separated from the unbound DNA probe by electrophoresis through a native 6% polyacrylamide gel in 0.5× TBE (Tris-Borate-EDTA buffer, pH 8.0). The digoxin-labeled oligonucleotide was detected with anti-digoxin antibody conjugated with alkaline phosphatase [32].

### 2.8. Statistical Analyses

Values will be expressed as means ± standard deviation (SD). Statistical evaluation was performed using Student's* t*-test or one-way ANOVA followed by Dunnett's test. A *P* value of <0.05 was considered significant.

## 3. Results

### 3.1. EGCG Increases HO-1 Expression in HASMCs

**Figure 2:**
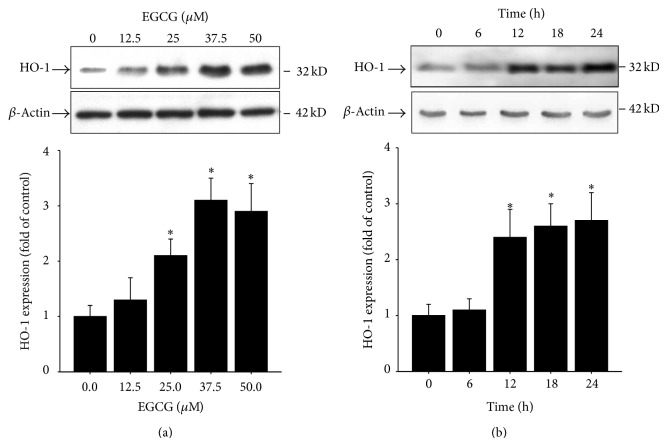
EGCG (a) dose- and (b) time-dependently induces HO-1 protein expression in HASMCs. Data are expressed as mean ± SD of three independent experiments. ^*^ *P* < 0.05 compared with medium alone condition.

To test the effects of EGCG on HO-1 expression in HASMCs, EGCG (12.5, 25, 37.5, and 50 *μ*M) was added in culture medium with HASMCs for 24 h, and Western blot was performed for HO-1 protein expression. As shown in Figure 2(a), EGCG dose-dependently increased HO-1 protein expression in HASMCs. Moreover, EGCG time-dependently (6, 12, 18, and 24 h) increased HO-1 protein expression in HASMCs.

### 3.2. Inhibitory Effect of EGCG on Proliferation and Oxidative Stress in HASMCs Induced by IL-1*β*

**Figure 3:**
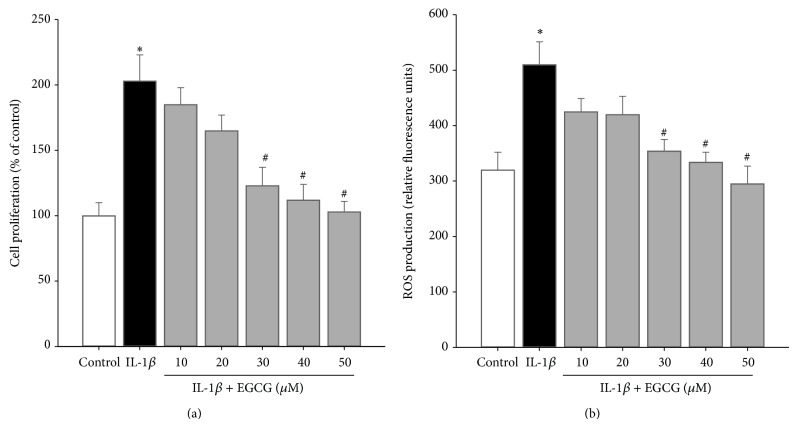
EGCG dose-dependently inhibits (a) cell proliferation and (b) ROS production in IL-1*β*-treated HASMCs. Data are expressed as mean ± SD of three independent experiments. ^*^ *P* < 0.05 versus control group; ^#^ *P* < 0.05 versus IL-1*β*-treated group.

To investigate whether EGCG inhibits the proliferation of HASMCs induced by inflammatory cytokine IL-1*β*, HASMCs cells were incubated with IL-1*β* (50 ng/mL) with EGCG (10–50 *μ*M) for 24 h, and BrdU incorporation assay was performed. EGCG had a dose-dependent effect to reduce proliferation of IL-1*β*-treated HASMCs. The significance was found in 30, 40, and 50 *μ*M concentration (Figure 3(a)).

Flow cytometric analysis was then used to determine whether the EGCG-induced cell growth inhibition was due to arrest at a specific point of the cell cycle. As shown in Table 1, flow cytometric analysis of the DNA content in HASMCs showed that EGCG treatment caused a significant increase in the percentage of cells in G_0_/G_1_ phase and a significant decrease in the S populations relative to IL-1*β*-treated cells.

To directly evaluate the effect of EGCG on ROS production, we evaluated ROS level in IL-1*β*-treated HASMCs. As shown in Figure 3(b), treatment with IL-1*β* (50 ng/mL) for 30 min caused a higher increase of fluorescence compared with control cells. However, pretreatment of HASMCs for 30 min with EGCG (10–50 *μ*M) dose-dependently and significantly inhibited IL-1*β*-induced ROS generation.

In addition, Table 2 shows that EGCG treatment caused a significant increase of GSH content and a significant decrease of TBARS content relative to IL-1*β*-treated cells.

### 3.3. EGCG Attenuates IL-1*β*-Induced Proliferation via HO-1

**Figure 4:**
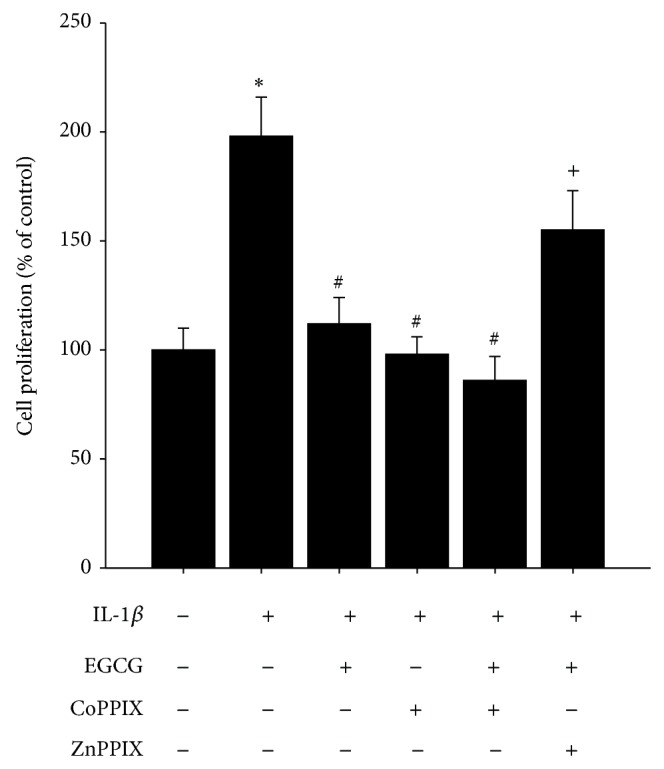
CoPPIX and ZnPPIX modulate proliferation inhibitory effect of EGCG in IL-1*β*-treated HASMCs. Data are expressed as mean ± SD of three independent experiments. ^*^ *P* < 0.05 versus control group; ^#^ *P* < 0.05 versus IL-1*β*-treated group; ^+^ *P* < 0.05 versus EGCG and IL-1*β*-treated group.

We assayed the effect of HO-1 expression on IL-1*β*-induced cell proliferation. As shown in Figure 4, we found that the HO-1 inducer CoPPIX decreased IL-1*β*-induced cell proliferation, whereas the HO-1 enzyme inhibitor, ZnPPIX, significantly reversed EGCG-caused growth inhibition in IL-1*β*-treated HASMCs.

### 3.4. EGCG Activates Nrf2 in HASMCs

**Figure 5:**
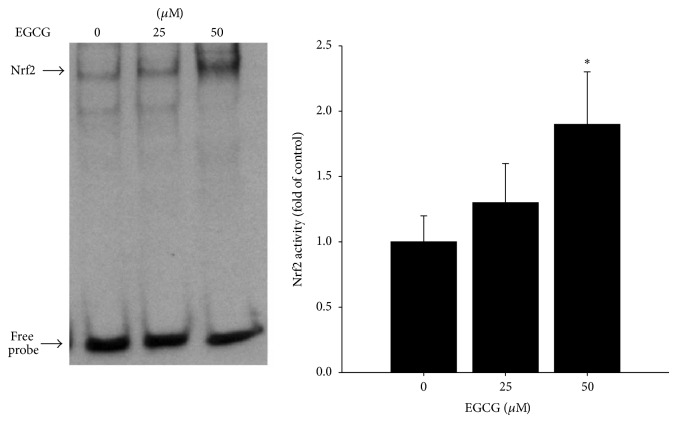
Effect of EGCG on Nrf-2 activation. Data are expressed as mean ± SD of three independent experiments. ^*^ *P* < 0.05 versus control group.

Activation of Nrf2 increases Nrf2 accumulation in the nucleus. It has been suggested that the regulation of Nrf2 transcriptional activation of HO-1 relies on subcellular distribution rather than induction of this transcription factor through* de novo* synthesis [31, 33]. Thus, we determined nuclear activation of Nrf2 in both control and EGCG-treated cells. As shown by EMSA in Figure 5, EGCG treatment significantly activated Nrf2 transcription activities through binding specifically to the ARE found in the promoters of target genes.

## 4. Discussion

The present study showed that, for the first time, green tea EGCG attenuated IL-1*β*-induced proliferation of HASMCs via HO-1-related mechanisms. EGCG treatment induced the arrest of cell cycle progression and increased ROS production as results in GSH deficiency and lipid peroxidation in VSMCs. Our data also suggest that Nrf-2 mediated HO-1 expression may play a pivotal role for the antiproliferative effect of EGCG on VSMCs.

Atherosclerosis causes clinical diseases through luminal narrowing of the brain (ischemic stroke), heart, and peripheral vessels. Oxidative stress contributes to the progression of cerebrovascular disease. It can induce important processes leading to cerebral aneurysm formation including direct endothelial damage as well as smooth muscle cell phenotypic switching to a proliferative and inflammatory phenotype [34]. Epidemiological studies indicated that regular consumption of green tea is associated with a reduced risk of coronary heart disease [35, 36].* In vitro* investigations have indicated that green tea polyphenolic compounds are able to inhibit several key events of the atherogenic process such as proliferation and migration of VSMCs by redox-sensitive mechanisms. EGCG-mediated HO-1 induction has been shown to occur in endothelial cells [31, 37–39], epithelial cells [40], neurons [41], kidney [42, 43], and pulmonary cells [44–46]. In the present study, the antioxidant properties of EGCG were further reported to protect against cardiovascular diseases by inhibiting inflammatory cytokine-induced VSMC proliferation via HO-1 induction. Thus, our results elucidated the relationship with inflammation, ROS, and regulation of atheroprotective genes HO-1 and how the regulation of these activities by EGCG can lead to a prevention of vascular diseases.

During atherogenesis, chronic inflammation and oxidative stress play a crucial role in VSMC activation. To eliminate the oxidative stress, endogenous antioxidant enzymes including HO-1 induction are evoked. The beneficial effects of HO-1 upregulation have been reported in a series of animal models [47]. Induction HO-1 induction is mediated by various mechanisms, including the blockade of immune response and increased production of carbon oxide [48]. HO-1 protects VSMCs from oxidative damage and against VSMC proliferation [49, 50]. In the present study, EGCG dose- and time-dependently induced HO-1 expression in HASMCs. Consistent with our results, EGCG also increases HO-1 expression and shows anti-inflammatory capacity in various cells such as endothelial cells [31, 37, 39] and monocytic cells [51]. All these findings suggest that EGCG is a strong inducer of HO-1 and such induction may be independent of various cell types.

Cell cycle is a major convergent point in VSMC proliferation. This process is controlled by multiple protein kinases and regulatory cyclins [52]. Negative regulators of the protein kinases and cyclins, therefore arresting the cell cycle at G_0_/G_1_ phase [48]. In the present study, EGCG inhibited DNA synthesis and arrested the cell cycle at G_0_/G_1_ phase. Thus, the protein levels of negative regulators of the protein kinases and cyclins need to be further investigated. Nevertheless, based on these findings, it is reasonable to speculate that the inhibitory effects of EGCG on VSMC proliferation are mediated by the induction of a cell cycle arrest.

Oxidative stress is defined as the imbalanced redox status in which prooxidants overwhelm antioxidant capacity, resulting in increased production of ROS. ROS have been implicated in the pathogenesis of virtually every stage of atherosclerosis [7, 8, 53]. ROS, especially superoxide anion and hydrogen peroxide, are important signaling molecules in cardiovascular cells. ROS participate in growth, apoptosis, and migration of VSMCs; these events play important roles in vascular diseases, suggesting that the sources of ROS and the signaling pathways that they modify may represent important therapeutic targets [54]. Numerous studies have shown that ROS influence cellular processes in vascular remodeling by turning on several intracellular signaling cascades. ROS potently activate mitogen-activated protein kinase members important in cell growth and differentiation, which induce expression of proinflammatory genes that play a role in the vascular inflammation associated with hypertension and atherosclerosis [54]. The present study provides the direct evidences that EGCG could maintain the concentrations of intracellular antioxidants found in biological systems. EGCG maintained GSH amounts in inflammatory cytokine-stressed HASMCs resulting in decreased TBARS.

Advances in signal transduction network indicate that a battery of redox-sensitive transcription factors, such Nrf2, and their upstream kinases play an important regulatory role in HO-1 gene induction [55]. The transcription factor Nrf2 plays an essential role in the ARE-mediated expression of phase II detoxifying and antioxidant enzymes and in the activation of other stress inducible genes. These genes may play a vital role in the prevention of cell dysfunction as a result of free radical production [56]. In the present study, Nrf2 was found to be markedly induced in HASMCs exposed to EGCG. The translocation of Nrf2 into the nucleus with treatment of EGCG was also found to be associated with increases in its ARE transcriptional activity. Our data therefore suggest that EGCG may induce many ARE-associated genes via its potent induction of Nrf2.

## 5. Conclusions

In summary, our results demonstrated that (1) EGCG dose- and time-dependently induces HO-1 expression in HASMCs; (2) EGCG dose-dependently inhibits IL-1*β*-induced HASMC proliferation and cell cycle arrest; (3) EGCG dose-dependently reduces IL-1*β*-induced oxidative stress in HASMC; and (4) Nrf-2 mediated HO-1 expression may play a pivotal role for the antiproliferative effect of EGCG on VSMCs. Thus, EGCG may likely fulfill the definition of a pharmacological preconditioning agent for preventing cerebrovascular and cardiovascular diseases.

## Figures and Tables

**Table 1:** Effect of EGCG on the cell cycle distribution in IL-1*β*-treated HASMCs.

Phase	IL-1*β*	EGCG + IL-1*β*	*P* value
Sub-G_1_	2.2% ± 1.1%	3.7% ± 1.2%	0.186
G_0_/G_1_	54.2% ± 7.9%	75.4% ± 8.3%	0.033∗
S	31.6% ± 5.3%	11.3% ± 4.3%	0.007∗
G_2_/M	12.0% ± 2.3%	9.6% ± 2.5%	0.288

^*^ *P* < 0.05.

**Table 2:** The GSH and TBARS levels in EGCG and IL-1*β*-treated HASMCs.

	IL-1*β*	EGCG + IL-1*β*	*P* value
GSH (nmol/mg protein)	27.3 ± 2.4	42.5 ± 3.5	0.003∗
TBARS (nmol/mg protein)	7.2 ± 1.4	3.4 ± 1.8	0.045∗

^*^ *P* < 0.05.
